# Data of furfural adsorption on nano zero valent iron (NZVI) synthesized from Nettle extract

**DOI:** 10.1016/j.dib.2017.11.035

**Published:** 2017-11-11

**Authors:** Mehdi Fazlzadeh, Mohammad Ansarizadeh, Mostafa Leili

**Affiliations:** a Department of Environmental Health Engineering, School of Public Health, Ardabil University of Medical Sciences, Ardabil, Iran; ^b^Department of Environmental Health Engineering, Mamasani Higher Educational Complex, Shiraz University of Medical Sciences, Shiraz, Iran; ^c^Department of Environmental Health Engineering, Research Center for Health Sciences, Hamadan University of Medical Sciences, Hamadan, Iran

**Keywords:** Green synthesis method, Furfural, Nettle zero valent iron nanoparticles (NNZVI), Low cost adsorbents

## Abstract

Among various water and wastewater treatment methods, adsorption techniques are widely used to remove certain classes of pollutants due to its unique features. Thus, the aim of this data article is to synthesize zero valent iron nanoparticles (NZVI) from Nettle leaf extract by green synthesis method as an environmentally friendly technique, and to evaluate it's efficiency in the removal of furfural from aqueous solutions. The data of possible adsorption mechanism and isotherm of furfural on the synthesized adsorbent are depicted in this data article. The data acquired showed that the adsorption trend follows the pseudo-second order kinetic model and that the Langmuir isotherm was suitable for correlation of equilibrium data with the maximum adsorption capacity of 454.4 mg/g. The information of initial furfural concentration, pH, adsorbent dosage and contact time effects on the removal efficiency are presented. Considering the findings data, the developed nanoparticle from Nettle leaf extract, as a low cost adsorbent, could be considered as promising adsorbent for furfural and probably similar organic pollutants removal from aqueous solutions.

**Specifications Table**

**Table:** 

Subject area	Environmental Engineering
More specific subject area	– Industrial effluent treatment – Wastewater technology
Type of data	Tables, Figures, Images and Text file
How data was acquired	– Nettle extract was used to synthesize novel nano zero-valent iron (NNZVI). – Batch experiments were performed to collect the data of the influence of contact time and pH on furfural removal. – Transmission electron microscopy (JEOL JEM 1200 EX Mk 2), Philips X’Pert Pro instrument (Netherlands), pH meter (Sense Ion 378, Hack), double beam spectrophotometer (Model lambda 25- Perkin Elmer Company) and Eppendorf versatile 5810 series centrifuge were used. – The obtained data were analyzed using appropriate equations and isotherm models.
Data format	Analyzed
Experimental factors	The data of effects of main experimental parameters including contact time and solution pH were acquired.
Experimental features	Adsorption of furfural from aqueous solutions using nano zero valent iron (NZVI) that prepared via green synthesis method from Nettle extract has be studied.
Data source location	Hamadan city, Hamadan province, Iran
Data accessibility	Data are available in article

**Value of the data**

• This data offer an environmentally friendly method for preparation of adsorbent from Nettle leaf extract.
• The removal of furfural from aqueous solution was examined using a synthesized novel green adsorbent.
• Data show that the developed adsorbent has high potential for the removal of furfural from aqueous solution.

## 1 Data

**Fig. 1:**
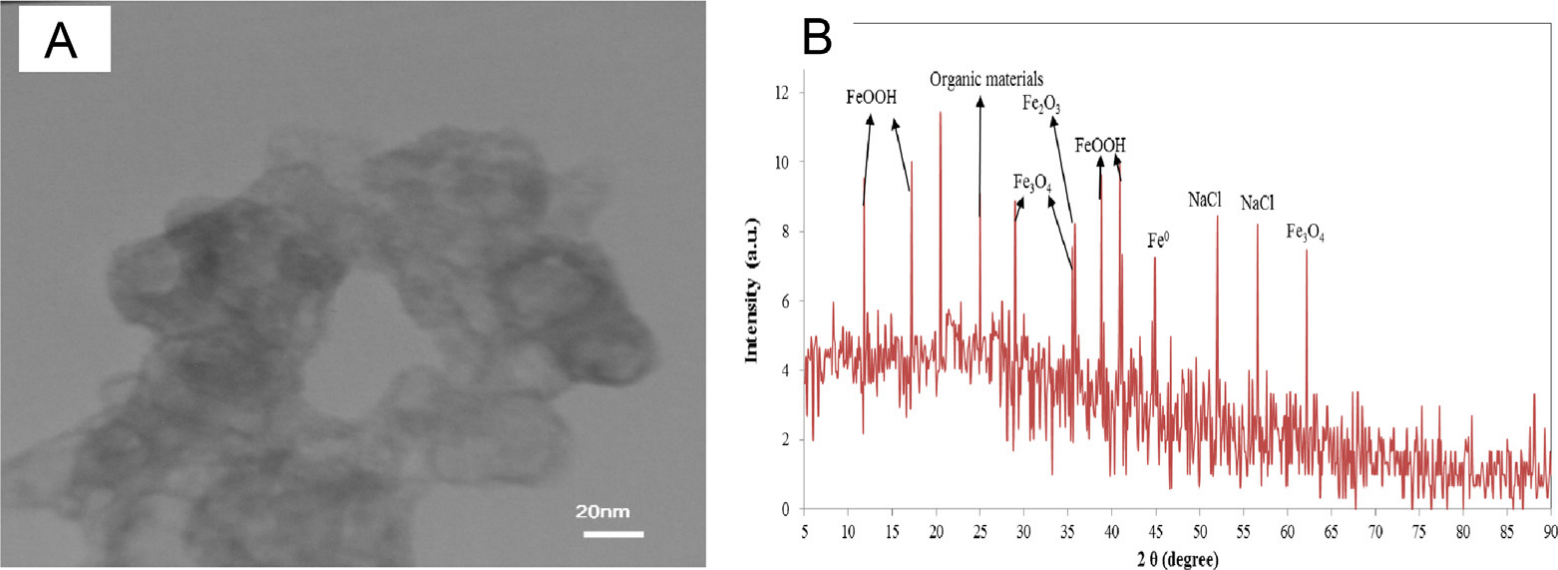
(a) TEM image of the synthesized NNZVI, and (b) XRD patterns of iron nano-impregnated particles.

**Fig. 2:**
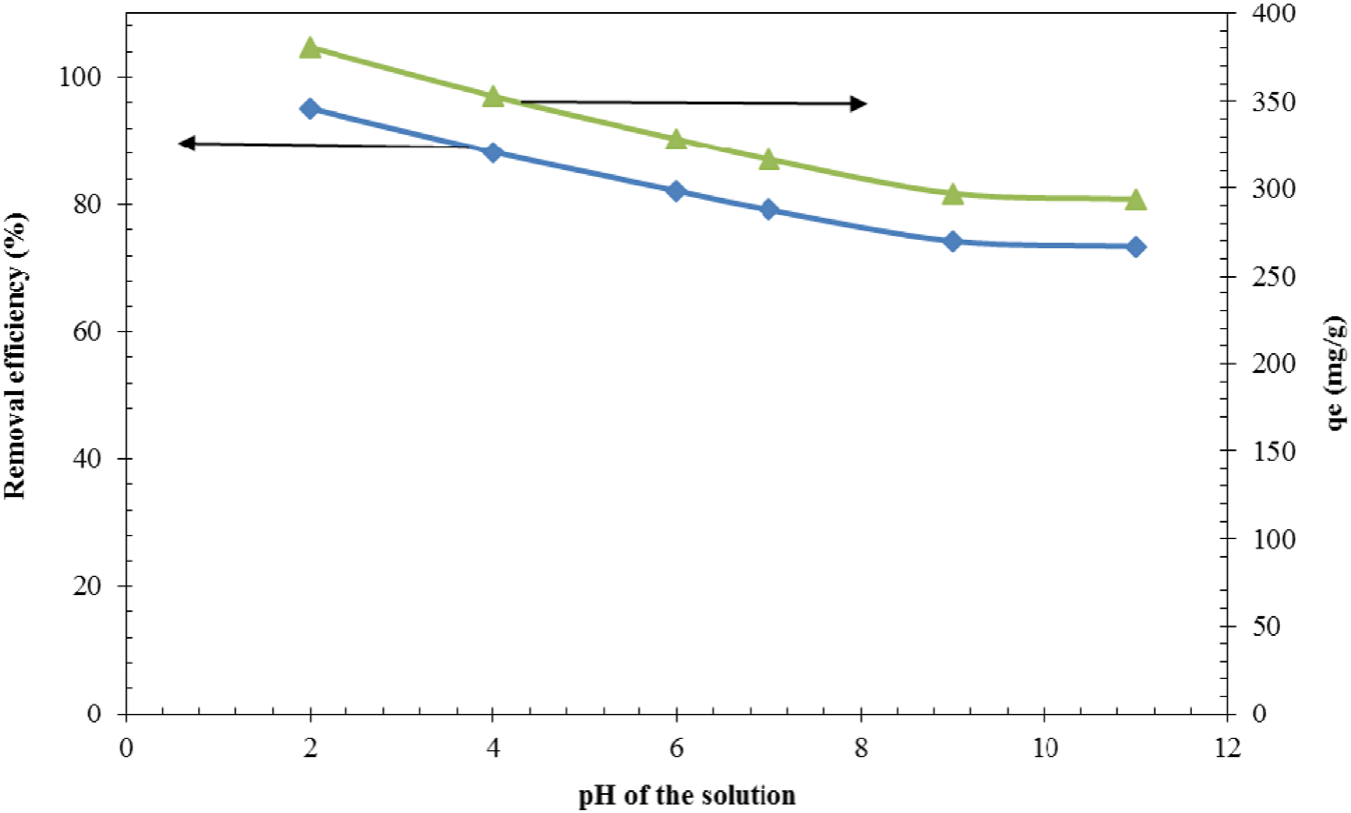
Effect of pH on furfural adsorption onto NNZVI (*C*_0_ = 200 mg/L, adsorbent dose =0.5 g/L, contact time = 50 min, shaking speed = 200 rpm at room temperature).

**Fig. 3:**
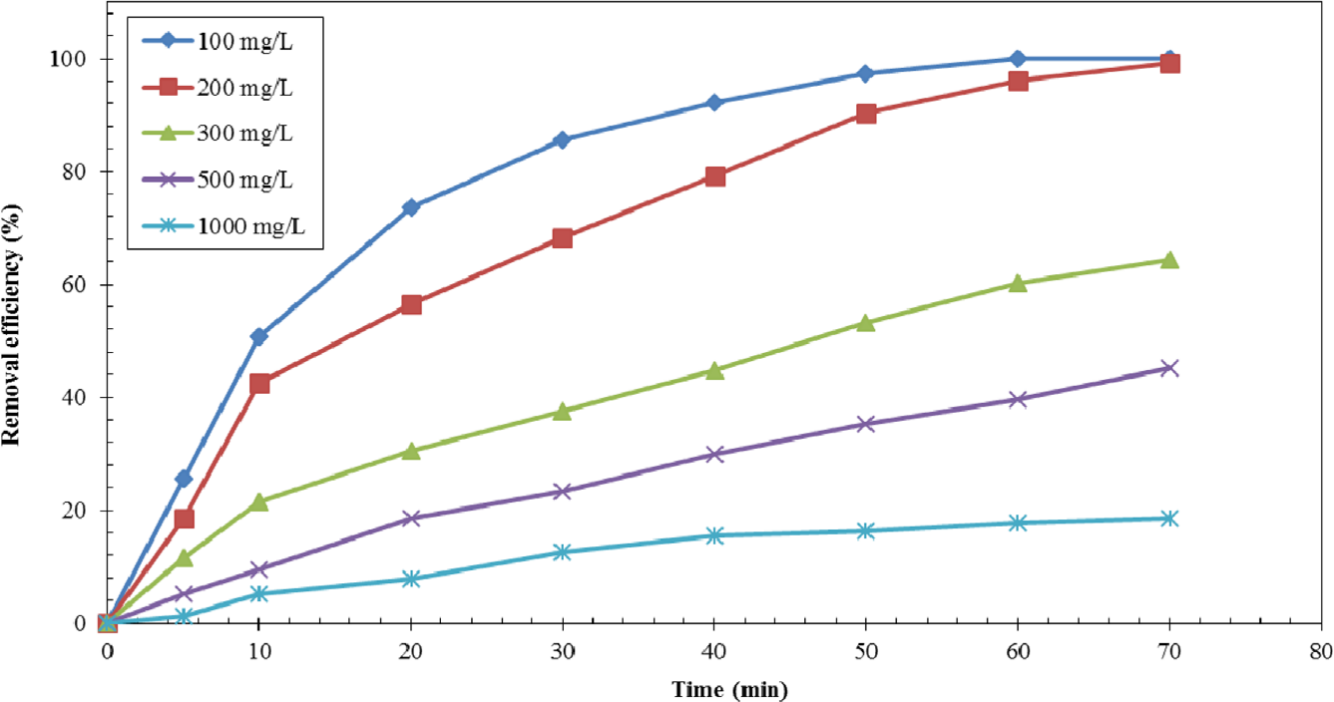
Effect of contact time on furfural adsorption onto NNZVI with various initial concentrations (adsorbent concentration = 0.5 g/L, pH =2, shaking speed = 200 rpm at room temperature).

The Nettle leaf, as an abundant local plants in Ardabil province, northwestern Iran, used in this study to prepare zero-valent iron nanoparticles (NZVI). Transmission electron microscopy (TEM), and Philips X’Pert Pro instrument (the Netherlands) were used to get particle sizes and XRD patterns of the synthesized nanoparticles, respectively. The obtained data are shown in Fig. 1(a) and (b). The effects of contact time and solution pH on removal efficiency data are presented in Fig. 2, Fig. 3. The kinetic and isotherm data are also shown in Table 1, Table 2.

**Table 1:** Parameters of the most common models applied to the adsorption kinetics of furfural onto synthesized NNZVI [1].

Adsorbent	*C*_0_ (mg/L)	*q*_e,exp_ (mg/g)	Pseudo-first-order			Pseudo-second-order		
			*K*_1_ (min^-1^)	*q*_e_ (mg/g)	*R*^2^	*K*_2_ (g/mg-min)	*q*_e_ (mg/g)	*R*^2^
NNZVI	100	200	0.131	512.8	0.845	0.00055	222.2	0.980
	200	400	0.059	445.8	0.944	0.00023	434.8	0.970
	300	340	0.061	395.4	0.933	0.00029	384.6	0.963

**Table 2:** Langmuir and Freundlich isotherm models used in this dataset to modeling furfural adsorption onto NNZVI [2].

**Adsorbent / Isotherm**	**Langmuir**			**Freundlich**		
	*q*_max_ (mg/g)	*K*_L_ (L/mg)	*R*^2^	*K*_f_ (mg/g) (mg/L)*n*	*n*	*R*^2^
**NNZVI**	454.5	0.234	0.995	389	65.5	0.261

## 2 Materials and methods

### 2.1 Materials

All chemicals used in the experiments were high purity analytical grade and purchased from Merck Co. Germany, and are used without further treatment. Aqueous solutions of furfural with desired concentration for the batch experiments were prepared by serial dilution of a 1% stock furfural solution [3].

### 2.2 Green synthesis of NZVI from Nettle extracts

Nettle leaves was purchased from local market and washed several times with double deionized water to remove any dust and dried. Briefly, 60 g/L of the leaves of the plant was boiled at 80 °C for 1 h. After about 1 h stagnant time to precipitate the extract, the supernatant was filtered by a vacuum pump. 0.1 M FeCl_2_.4H_2_O solution was prepared by adding 19.9 g of solid FeCl_2_.4H_2_O into 1 l of deionized water. This solution was then added into the filtered supernatant that prepared in previous step in the ratio 2:3 [4]. At this time, a black colored precipitate was appeared which show the formation of NNZVI. The formed nanoparticles were then separated by evaporation on a hot plate surface and collected by washing several times with deionized water and placed in nitrogen gas to avoid oxidation and regarded as NNZVI [5] and used as adsorbent in the experiments.

### 2.3 Determination of furfural content and adsorption–desorption experiments

A colorimetric method was used to analyze the furfural concentration of the samples. Furfural was measured at a wavelength of 277 nm using a double beam spectrophotometer (Model lambda 25- Perkin Elmer Company) [6].

Batch system was used to collect the required data and adsorption experiments were performed in 250 mL Erlenmeyer flasks. Determinate dose of NNZVI was added into the Erlenmeyer flask and was shaked immediately in regulated speed by shaker. After desired contact time, to separate adsorbents from aqueous solution, the samples were filtered through Whattman filter paper (0.2 µm) and then centrifuged (Eppendorf versatile 5810 series centrifuge) to simply calculate corresponding efficiency using Eq. (1) [7]:

(1) $$R(\%)=(1\;-\;\frac{C_{t}}{C_{0}})\times 100$$

where *C*_0_ and *C*_t_ are respectively the initial and final concentrations of furfural.

For kinetics studies, 0.5 g of adsorbents was contacted with 250 mL of furfural solutions in a beaker as carried out in previous step. In all kinetic experiments, the solution pH was kept at optimum value of ≈ 2, which was adjusted using 0.1 N HCl or NaOH solutions and measured using a pH meter (Sense Ion 378, Hack). The furfural adsorption capacities at equilibrium, *q*_e_ (mg furfural/g adsorbents), was determined using Eq. (2):

(2) $$q_{e}=\frac{{(C}_{0}\;-{\;C}_{t})\times V}{m}$$

where *C*_0_ and *C*_t_ are initial and final concentrations of furfural (mg/L), *V* is the volume of solution (L), and *m* is the nanoparticles mass (g) as dry.
