# Supplementary material for: Data of furfural adsorption on nano zero valent iron (NZVI) synthesized from Nettle extract
Source: Data Brief. 2017 Nov 11;16:341–5. doi: 10.1016/j.dib.2017.11.035 (PMC5711664; doi:10.1016/j.dib.2017.11.035)
Supplement: Supplementary file 1 — Supplementary material [file mmc1.doc]

**Conflict of interest**

The authors declare that they have no conflict of interest.

Regards,

Mostafa Leili

Corresponding author
